# DIET AND NUTRITION: White House Proposes Healthy Food Financing Initiative

**DOI:** 10.1289/ehp.118-a156

**Published:** 2010-04

**Authors:** David C. Holzman

**Affiliations:** **David C. Holzman** writes on science, medicine, energy, economics, and cars from Lexington and Wellfleet, MA. His work has appeared in *Smithsonian*, *The Atlantic Monthly*, and the *Journal of the National Cancer Institute*

The Obama administration announced in February a $400 million initiative it hopes will lure retailers of healthy foods into the so-called food deserts of America. The program, proposed as part of the fiscal year 2011 budget, aims to boost public health by eliminating urban and rural food deserts within 7 years.

The term “food desert” refers to areas that, although often served by fast food restaurants and convenience stores, lack easy access to affordable fruits, vegetables, whole grains, low-fat milk, and other foods that make up the full range of a healthy diet. About 23.5 million people—including 6.5 million children—live in low-income areas that are more than 1 mile from a supermarket, according to the June 2009 report *Access to Affordable and Nutritious Food: Measuring and Understanding Food Deserts and Their Consequences* by the U.S. Department of Agriculture (USDA).

The new Healthy Food Financing Initiative (HFFI) would be administered jointly by the Departments of Health and Human Services, Agriculture, and the Treasury, and would dovetail with Michelle Obama’s recently announced “Let’s Move” campaign to end childhood obesity within a generation. It would emphasize provision of fresh produce. And although the primary goal is nutrition, it would also seek to “create jobs and economic development, and establish market opportunities for farmers and ranchers,” said agriculture secretary Tom Vilsack at the program’s announcement.

That is what a Pennsylvania program, a model for the HFFI, has done, says Ann Wright, the USDA deputy undersecretary for marketing and regulatory programs. Launched in 2004, the Pennsylvania Fresh Food Financing Initiative has opened about 80 stores ranging from small mom-and-pop grocers to large full-service supermarkets, providing food for around 400,000 people, says John Weidman, deputy executive director of The Food Trust (TFT), 1 of 3 nonprofits managing the program. The program also has provided jobs for 4,800 people, he says.

The public health problem these programs address is obvious but imprecisely defined. Many of the highly processed, fat- and sugar-rich foods sold at convenience stores and fast food restaurants are implicated in cardiovascular disease, diabetes, and cancer. Several studies have reported that easy access to healthy foods and limited access to convenience stores is associated with healthier eating and reduced obesity, according to a review by Nicole Larson et al. in the January 2009 issue of the *American Journal of Preventive Medicine*.

However, the actual health toll from living in a food desert environment has not been tabulated in a peer-reviewed study. Moreover, the only 2 studies that examined diets before and after grocery stores were installed in food deserts—rather than comparing neighborhoods with grocery stores to similar neighborhoods without—are not encouraging, says Steven Haider, an associate professor of economics at Michigan State University. Neil Wrigley et al. wrote in volume 35, issue 1 (2003) of *Built Environment* that people consumed an extra half a serving of fruit and/or vegetables daily, while Steve Cummins et al. reported no change in the Winter 2005 issue of *Planning Healthy Towns and Cities*. And global nutrition professor Barry Popkin of the University of North Carolina at Chapel Hill says a January 2009 workshop he chaired at the Institute of Medicine on the public health effects of food deserts “could find no evidence that adding new retail stores to depressed areas changed what people consumed.”

Meanwhile, Amy Lanou, an assistant professor of health and wellness at the University of North Carolina at Asheville, argues that education, a demand-side measure, is needed to maximize the benefits of the HFFI. (Demand-side measures are measures that boost peoples’ desire to purchase healthy food. Their converse, supply-side measures, are those, such as grocery stores, that make it more available.) Wright says the USDA has been promoting better nutrition in schools through agency programs and support for local efforts to bring healthy food into the school cafeteria. But while school lunches must meet specific nutritional requirements, Lanou says schools “can sell almost anything à la carte,” giving kids the option of eating unhealthily if they can afford it.

Mari Gallagher, whose eponymous research and consulting company has studied food deserts extensively in Chicago and other cities, says it’s not enough to live near healthy food outlets. People who are time-stressed—working multiple jobs, for instance, or commuting on several different transit lines—will travel half a mile for junk food rather than a mile for healthy food, she says.

If access to affordable wholesome food alone does not alter eating habits, perhaps other factors will. In the 8 March 2010 issue of *Archives of Internal Medicine*, Popkin showed that localized hikes in fast food prices over a 20-year period tracked with reduced risk of obesity and diabetes in affected communities. Another study finds cultural sensitivity is important in promoting healthy eating. “Our qualitative research in New York City suggests that Hispanic immigrants conceptualize ‘healthy foods’ more in terms of freshness and local origin than in terms of nutritional content,” says Andrew Rundle, an associate professor of epidemiology at the Mailman School of Public Health, Columbia University. This is consistent, he says, with initial findings in Hispanic neighborhoods that access to farmers’ markets was a better predictor of produce consumption than access to supermarkets.

Gallagher warns that determining which locales have the greatest need for subsidies, as well as keeping politics from affecting the flow of money, will be challenging. It is important to “make sure we’re armed with neutral data that directs the flow of resources,” she says. Nonetheless, she says, “I’m very thrilled [about the proposed initiative]. We think this is needed. We encourage the administration to disburse these funds with the best data and methods so we get the highest public health return for the investment.”

## Figures and Tables

**Figure:**
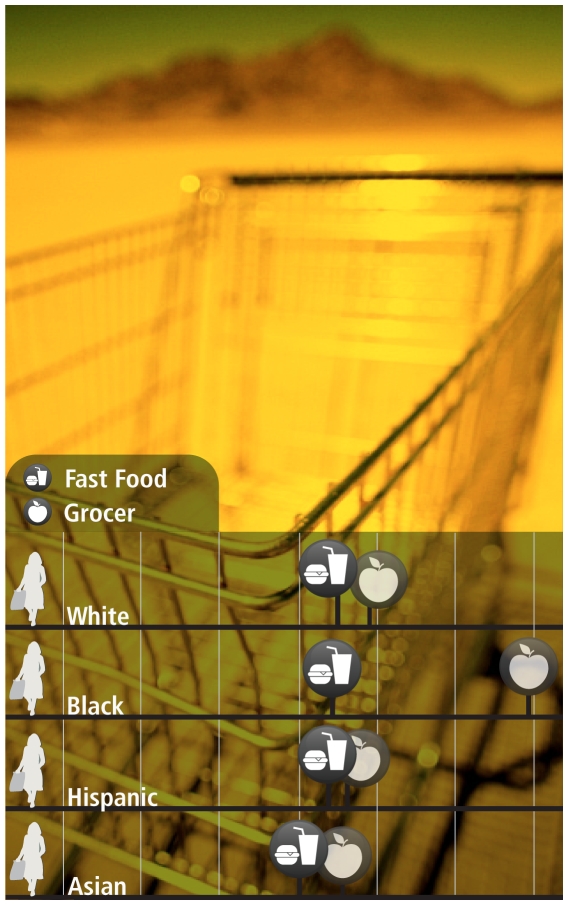
**In a study of Chicago food deserts, black residents had to travel farther than other racial groups to reach a grocery store but not a fast food restaurant.** Distance shown in tenths of a mile. Adapted from Mari Gallagher Research & Consulting Group. 2006. Examining the impact of food deserts on public health in Chicago.

